# Singular Case of Death Occurring Twenty-two Days after Instrumental Delivery

**Published:** 1856-08

**Authors:** J. M. Gemmill

**Affiliations:** Alexandria, Pa.


*Singular Case of Death occurring twenty-two days after instrumental delivery.* By J. M. Gemmill, M. D., of Alexandria, Pa.

The following case occurred in my practice, and as the cause of the fatal result is somewhat uncertain, I submit it to the profession, in order that attention may be directed to the cause of death in similar cases.

At two o'clock, A. M., January 10, 1853, I was called to see Mrs. H., supposed to be in labor with her first child. I found her with some slight pains in the back, without any marked regularity. After waiting an hour, I examined per vaginam, and found the os uteri closed, and high up in the pelvis, without any sign of labor having commenced. I bled her about sixteen ounces, and remained a few hours, when the pains subsided.

I then left her, and was called again on the next morning (Tuesday) at four o'clock. I found her much as on the previous morning, except that she was now troubled with desire to urinate frequently, with inability to discharge more than a very small quantity at a time; but still sufficient to prevent any large accumulation in the bladder. As the pulse was full and strong I bled her a small quantity and gave her an anodyne. This relieved the pain and uneasiness, but on the next day the difficulty in urinating had increased, and the bladder was distended. On examining by the vagina, the head of the child was found low down in the pelvis, but the os uteri undilated. On introducing the common female catheter, the bladder could not be reached. I then took a flexible male catheter, and passed it up nearly the whole length, and through this a portion of the contents of the bladder was discharged. From Tuesday morning she had no pain except that produced by the distension of the bladder. On Thursday the condition of the patient was about the same as on the preceding day as it regards the urinary organs, and by the use of the M. catheter, the contents of the bladder were discharged. The os uteri was now fully dilated, and the distended membranes were felt just within the os externum. The head was low down, though still surrounded by the lips and neck of the womb, and yet she had no pains like those of labor.

She had by this time become feverish, and her feet were considerably swollen. With the hope of exciting labor pains, I now ruptured the membranes, and discharged a large quantity of very offensive liquor amnii. I left her with directions to send for me if labor should come on.

Having heard nothing from her, on the next day (Friday) I visited her and found her with hot skin, full, frequent pulse, swelled feet, great thirst, &c. The child's head now rested on the perineum, the bladder was distended, but still there was no labor pain. I now gave her ergot in full doses without any effect, and then determined to deliver with the forceps. After evacuating the bladder and rectum, the forceps were introduced and the child delivered without much difficulty. The placenta was detained by extensive adhesions and hour-glass contraction. To accomplish its delivery, the hand was introduced and the placenta peeled off by the fingers, and brought away on withdrawing the hand. This was effected without much loss of blood or any unusual difficulty. She was, however, much exhausted, and required some gentle stimulus to revive her. This was ordered, and the womb being well contracted, I left her for the night. On the following morning reaction was established; as she had passed no urine, the catheter was introduced, and the bladder emptied. The nurse was instructed in the use of the catheter, and directed to use it three or four times in twenty four hours, which she was able to accomplish without any trouble. On the next day she began to have irritative fever, which ran high, without any pain or tenderness in the abdominal or pelvic regions; the appetite failed, the bladder required the use of the catheter, and the vaginal discharge was very offensive. She was treated for these symptoms as circumstances seemed to demand, for about ten days, when they began to subside. The pulse came down from 120 or 130 to from 90 to 100; the appetite improved and convalescence seemed to be established. On the 27th of the month she was able to sit up, the appetite was good, and every thing progressed favorably, except that the bladder had not yet recovered the power of expelling its contents, and the bowels required an occasional teaspoonful of castor oil. She was taking quinia and iron by hydrogen, in small doses frequently repeated.

On Monday evening, January 31, I visited her, and found her pulse ninety in the minute, tongue clean, no pain or tenderness any where, the appetite good, and all the functions well performed, except that the urine had to be drawn off by the catheter, and the bowels did not move without medicine. As there had been no discharge from the bowels for several days, I directed her to take one teaspoonful of castor oil the next morning, if there should be no discharge in the mean time; and to continue the tonic medicine. At this visit she appeared to be entirely well, except the debility and the want of power in the bladder to expel its contents.

On Tuesday evening, about twenty hours from the time I left her, I was sent for to see her. On my arrival I found her pulse beating one hundred and sixty-five in a minute, great dyspnœa, and almost complete inability to talk from shortness of breath; and fainting on the slightest exertion. On examination I found there had been no hemorrhage; there was no abdominal distension, pain, or tenderness. I learned from the nurse that she had taken a teaspoonful of castor oil in the morning, which had moved her bowels twice freely by noon. At this time she felt quite well. About three o'clock she complained of being tired lying, and said she thought she would feel better if she sat up. This she had frequently done before. She was taken up and placed in an armed chair, but had only sat a few minutes when she fainted. She was laid down as soon as possible, but on emerging from the fainting fit, the above mentioned distressing symptoms presented themselves, and continued till death ensued, about five o'clock the following morning.

No post-mortem examination was permitted, although earnestly solicited. The question presented for solution is, What was the cause of death? I can conceive of no cause that will account for the symptoms presented, except the formation of a heart clot.

On the preceding evening her heart beat ninety in a minute, and her breathing was natural, and I have no doubt this continued to be the case until she was taken up and placed in the chair. Her husband stated that when he was in to dinner at noon, she appeared quite well, and the nurse said she continued so until the fainting fit occurred, immediately after which the symptoms above described made their appearance. What other cause could produce the great change in the heart's action, and the respiratory function ? I can conceive of none. The probability is that the fainting was caused by assuming the erect position soon after having had two free discharges from the bowels; and during the cessation of the heart's action the blood in one or more of its cavities coagulated in such quantity as to interfere with its functions to such a degree as to produce the symptoms presented, and ultimately death. In the absence of a post-mortem examination this seemed to me the most plausible explanation, and such was my diagnosis at the time.

The dilatation of the os uteri, and relaxation of the soft parts, without any thing like labor pain, is rather a singular feature in the case.

